# Recent progress in field-directed assembly of colloids in an evaporating droplet

**DOI:** 10.1016/j.mtbio.2025.102072

**Published:** 2025-07-08

**Authors:** Yongqing He, Jian Liu, Xukai Yang, Jianzhi Yang, Feng Jiao

**Affiliations:** a Department of Energy and Environment, Southeast University, Nanjing, 211189, China; b School of Chemical Engineering, Kunming University of Science and Technology, Kunming, 650500, China

**Keywords:** Evaporation, Droplets, Colloidal particles, Assembly

## Abstract

Evaporation-driven self-assembly of colloidal particles in a sessile drop can construct simple structures in various scenarios. However, the non-equilibrium self-assembly process, dominated by internal capillary flow and solute- or thermal-induced Marangoni flow, cannot guarantee the precise positioning of the particles. One can exert forces on individual suspended particles by introducing external field manipulation, which enables the construction of precise deposits and even 3D structures. Here, we review recent advances in field-directed assembly techniques, including acoustics, optics, electronics, and magnetics, where micro- and nano-particles are directly guided into targeted positions to form functional structures. These techniques are fundamental in analytical chemistry for their ability to achieve precise particle positioning, which is essential for developing sensitive and specific analytical methods. We first discussed the forces generated by these external fields and then summarized the directed assembly of colloidal particles, including its benefits and disadvantages for analytical chemistry applications. Furthermore, we examine the challenges and potential solutions for controlling colloidal particles and explore future research directions to advance the field of analytical chemistry further.

## Highlights

• Emphasis on advantages of directed assembly of colloidal particles in an evaporating drop.
• Four external manipulations for the deposition morphology of colloidal particles are mainly reviewed.
• Challenges and perspectives for the directed assembly of colloidal particles are proposed.

## Graphical abstract

**Figure:**
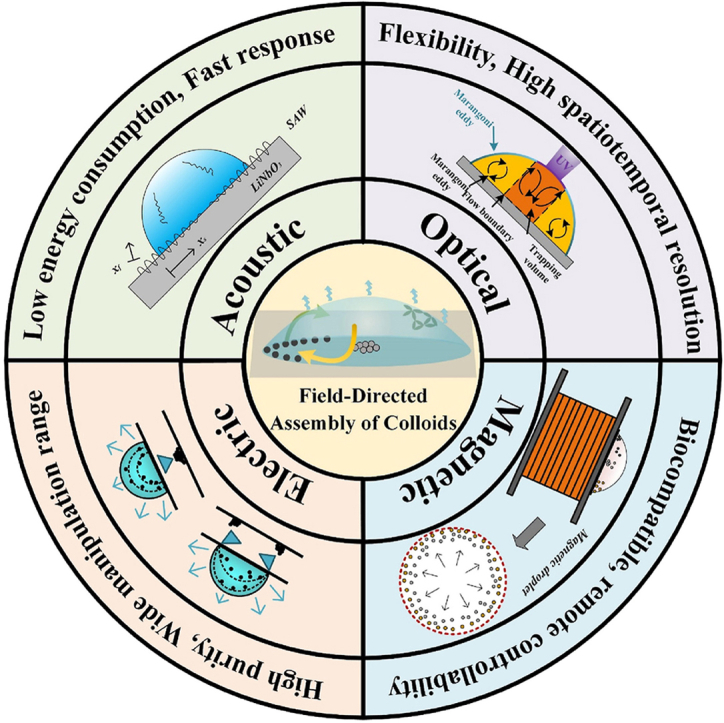

## 1 Introduction

Droplet evaporation is a ubiquitous process governed by complex fluid dynamics [[1], [2], [3]]. Among these phenomena, the evaporation-driven assembly of colloidal particles has garnered significant interest from researchers, due to its ability to organize micro- and nanoparticles into specific structures [4,5]. Nevertheless, the inherent stochasticity of this self-assembly process poses a fundamental challenge for analytical applications that demanddemanding multifunctional architectures, necessitating improved precision and integration density. Studies indicate that variations in evaporative flux at the gas-liquid interface during droplet evaporation govern internal flow dynamics, impacting assembly outcomes. Without external fields, colloidal particles in evaporating droplets spontaneously form ordered structures through self-assembly, driven by interactions such as hydrogen bonding [6], electrostatic forces [7], van der Waals interactions [8,9], charge compensation [10], and capillary effects [11,12]. This process can be widely applied in microfluidics [13], the development of biosensors [14,15], diagnostics [16], and multifunctional manufacturing [17]. However, self-assembly's reliance on dynamic internal factors [18,19], such as particle properties (composition, size, shape [20,21]), biological molecules (proteins, DNA), and solution conditions (pH, surfactant presence [22,23], and the concentration of components [24,25]), introduces unpredictability, limiting the development of its use in areas demanding high precision, such as advanced diagnostics or nanofabrication [26,27]. The self-assembly process is susceptible to subtle changes in the flow field within the droplet, which makes it less suitable for applications requiring high precision in analytical chemistry. Furthermore, the growing demand for complex, multifunctional structures across various fields highlights the need to overcome the limitations of self-assembly in flow field control and precise positioning. Consequently, this has spurred the development of field-directed assembly techniques to improve the precision and efficiency of the assembly process.

Evaporation-driven self-assembly is governed by capillary flows that radially transport colloidal particles to droplet peripheries, resulting in a coffee-ring deposition pattern that intrinsically limits structural uniformity [28]. The Coffee Ring Effect (CRE) compromises the accuracy of analytical testing by creating uneven particle distributions. For example, Gulka et al. [29] demonstrated that CRE in malaria biomarker assays results in inconsistent signal detection, thereby reducing diagnostic reliability. Similarly, uneven deposits in high-precision printing hinder pattern fidelity [20]. Field-directed assembly mitigates these issues by promoting uniform particle deposition. This inhomogeneity, dictated by unbalanced capillary forces and interfacial tension gradients, constrains the spatial fidelity required for precision analytical platforms [30]. To circumvent these limitations, field-directed assembly emerges as a critical strategy for controlling particle dynamics and overcoming stochastic flow instabilities, thereby enhancing analytical resolution. External fields modify assembly pathways by overriding equilibrium-driven interactions, enabling deterministic patterning of hierarchical architectures for biosensing and diagnostic interfaces [31]. It significantly enhances the controllability and precision of colloidal particle assembly, allowing the creation of functional materials with structures from one-dimensional to multi-dimensional. Such field-mediated control unlocks programmable synthesis of functional materials with tunable dimensionality (1D nanowires to 3D superlattices), bridging colloidal self-organization, and device-oriented engineering [29,[32], [33], [34]].

Standard methodologies for external field manipulation encompass surface acoustic waves (SAW) [35], optical fields [[36], [37], [38]], electrowetting (EW) [39], and magnetic fields [40,41]. These fields can either directly apply mechanical forces to colloidal particles or modulate the surrounding hydrodynamic environment, thereby indirectly governing particle dynamics. Such capabilities enable precise nanoparticle operations, including sorting, aggregation, and spatial organization, ultimately facilitating the fabrication of specific functional architectures [42]. Notably, Chen et al. [36] systematically reviewed light-directed colloidal assembly, emphasizing its fundamental distinctions from other field-mediated approaches. Each field modality exhibits inherent limitations contingent upon environmental conditions, particle characteristics, and operational parameters, necessitating careful consideration when selecting a technique. Advancing the practical application of field-directed assembly requires a systematic comparative analysis of the underlying manipulation mechanisms across different field modalities. Such analysis would establish a rigorous theoretical framework to guide their application-specific implementation. Future advancements in assembly methodologies will likely integrate multidisciplinary approaches to address the growing demand for enhanced complexity and precision in nanoscale manipulation processes.

Although directional assembly of nanomaterials has been extensively reviewed in static or bulk-phase systems [[43], [44], [45]], with in-depth discussions on molecular interactions, mechanisms of multiple external fields (electric, magnetic, optical, and fluidic fields), and their applications in sensing, electronics, catalysis, and biomedicine [[46], [47], [48]] research on field-directed assembly within the unique dynamic environment of evaporating droplets remains scarce, particularly in the context of microfluidic technology and analytical chemistry. Grzelczak et al. [43] examined the mechanisms of directional self-assembly mediated by molecular interactions and external fields (electric and magnetic fields), highlighting the pivotal role of templating in nanoparticle organization. Chai et al. [44] surveyed field-directed nanomaterial assembly driven by electric, magnetic, hydrodynamic, and optical fields, with critical analysis of technological applications in sensing platforms, electronic components, and nanoelectromechanical systems. Wang et al. [45] investigated the assembly dynamics of inorganic nanoparticles under electric, magnetic, and shear fields, exploring emergent applications that span catalysis, sensing technologies, and advanced energy storage systems. Fu et al. [46] reviewed stimulus-responsive plasmonic nano assemblies for biomedical applications, with a focus on biosensing platforms and photoactivated therapeutic systems. Jeon et al. [47] elucidated biologically inspired photonic materials, while Zhang et al. [48] investigated tunable plasmonic nanodevices, both emphasizing the manipulation of optical properties for biosensing and optical communication applications.

This review systematically outlines the fundamental physical principles of acoustic, optical, electrical, and magnetic fields, as well as the force field distributions they induce. It elucidates the differentiated mechanisms driving colloidal assembly and provides an in-depth analysis of the coupling dynamics between fields and particles. Through a critical evaluation of experimental data, supplemented by quantitative schematic diagrams and phase diagram analysis, it objectively compares the advantages and limitations of various field manipulation methods. It is worth noting that single-field strategies are often constrained by the complexity of colloidal interactions, field penetration depth, and the dynamic interplay of key evaporation parameters (such as interfacial tension and evaporation rate). Therefore, future research will shift toward hybrid field strategies, integrating complementary physical phenomena (such as light-electricity or sound-magnetic coupling) to achieve precise, high-fidelity control of colloidal structures at the nanoscale. In summary, this review not only outlines the transformative potential of field-directed colloidal assembly using evaporating droplets as a key platform, providing a roadmap for its applications in analytical chemistry and biomedical engineering (e.g., biosensing, diagnostics), but also identifies the core challenges-including scalable preparation, energy efficiency optimization, and precise control of complex solid/liquid/gas interfaces-that must be overcome for practical implementation.

## 2 Fundamental dynamics of field-directed colloidal assembly in evaporating droplets

**Fig. 1:**
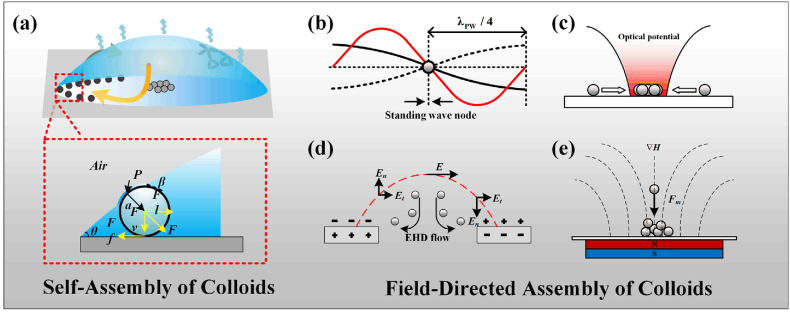
Schematic illustration of forces acting on colloidal particles in an evaporating droplet under various conditions. (a) Self-Assembly under Internal Stresses: In the absence of external forces, colloidal particles within an evaporating droplet experience internal stresses driven by evaporation-induced capillary flow and interparticle interactions. (b) Acoustic Field-Directed Assembly: Particles are manipulated by standing surface acoustic waves (SAWs) with a quarter-wavelength spacing ($\lambda_{PW}/4$); (c) Optical Field-Directed Assembly: A laser-induced optical potential gradient directs particle movement across the droplet surface; (d) Electric Field-Directed Assembly: An electric field, established between parallel electrodes, induces particle migration through electrophoretic ($E_{n}$) and dielectrophoretic ($E_{t}$) forces; (e) Magnetic field-directed assembly, in which the magnetic field pulls the magnetized colloidal particles.

The evaporation of a sessile droplet induces colloidal particle migration toward the contact line. This migration is governed by spatially heterogeneous evaporation rates and Marangoni-driven capillary flows, which collectively drive the self-assembly driving mechanism (Fig. 1a). By contrast, external fields-including acoustic, optical, electric, and magnetic fields-enable precise field-directed assembly through two distinct pathways: (i) direct exertion of forces on particles via field-particle interactions and (ii) controlled modulation of hydrodynamic flows within the evaporating droplet. Furthermore, the final deposition morphology depends critically on substrate wettability and interparticle interactions (such as electrostatic repulsion or depletion attraction). This direct influence underpins its relevance for analytical chemistry applications, ranging from biosensing platforms and advanced particle characterization techniques.

### 2.1 Acoustic Field-Directed Assembly

Acoustic field-directed assembly primarily utilizes two forces to control particle deposition within the droplet: the acoustic radiation force (ARF) and acoustic streaming flow (ASF). The ARF arises from momentum transfer in stationary sound waves (Fig. 1b) and guides particles towards pressure nodes. The magnitude of the ARF is proportional to the radius of colloidal particles. By adjusting the input frequency of sound waves, particles can be driven to specific positions in the droplet, counteracting capillary flow and enabling precise particle positioning. The ARF can be approximated using a simplified expression (Eq. (1)) [35,49]. The ASF is a secondary flow phenomenon resulting from the bulk fluid motion induced by the acoustic field. Also, it exerts forces that influence particle transport and deposition within the droplet [50].

(1) $$\begin{array}{c} F_{AR,L}=2\pi \rho {\left(\frac{2\pi f}{c_{f}}\right)}^{4}R^{6}{\left|\dot{\xi }\right|}^{2}\frac{1+\frac{2}{9}{\left(1-\frac{\rho }{\rho_{P}}\right)}^{2}}{{\left(2+\frac{\rho }{\rho_{P}}\right)}^{2}} \end{array}$$

Where, ρ, fluid density; $\rho_{p}$, the density of internal particles, *f*, the frequency of the incident acoustic field; $c_{f}$, the speed of acoustic waves in a fluid. The ARF predominantly governs suspended particles within the droplet, enabling size-dependent separation by exerting forces that are proportional to the particle radius and acoustic frequency. Traveling surface acoustic waves (TSAWs) provide an established framework for modeling ARF effects on rigid particles, where King [51] quantitatively defines the dependencies on particle radius (*R*), fluid density ($\rho_{f}$), and acoustic parameters. Concurrently, ASF generates convective flows that complement ARF, with its relative contribution often secondary in current models.

### 2.2 Optical field-directed assembly

The optical field-directed assembly utilizes focused light to precisely manipulate colloidal particles within evaporating droplets. This technique operates through three primary mechanisms [[52], [53], [54]]: (1) photothermal effects at the droplet surface or interior inducing Marangoni flows. These flows, resulting from surface tension gradients caused by laser-induced temperature variations, drive fluid from regions of low surface tension to high surface tension, thereby indirectly guiding particle motion [55]; (2) substrate-mediated light absorption, generating thermal gradients and convective flows that influence assembly; and (3) direct optical forces (radiation and gradient forces) acting on photosensitive particles. The direct optical forces (Fig. 1c), stemming from momentum transfer [56], drive particles toward focal regions, with radiation pressure quantifiable via simplified models (Eq. (2)) [57]:

(2) $$\begin{array}{c} F_{rad_{pressure}}=\frac{2n_{1}P}{c}{\left(\frac{a}{\omega }\right)}^{2}Q^{*} \end{array}$$

where P is the power of the laser, n_1_ is the refractive index of the medium, ω is the radius of the beam, c is the speed of light, a is the radius of the particles; $Q^{*}$ is the conversion factor, which expresses the efficiency of the transfer of the radiation pressure of the light-captured object

Multiple factors, including the power and wavelength of the optical field, the irradiation intensity, the size and material of both the colloidal and photosensitive particles and the properties of the surrounding fluid medium, influence the optical assembly. Directional assembly of colloidal particles occurs only when the optical force exceeds a specific threshold value, overcoming the surface adhesion, elasticity, and medium-induced static friction experienced by the particles [57].

### 2.3 Electric field-directed assembly

Electric field-directed assembly employs electrophoretic (*F*_*EP*_) and dielectrophoretic (*F*_*DEP*_) forces to manipulate nanoparticles within droplets, with approaches categorized as intrusive (direct electrode contact) or non-intrusive (external field application) (Fig. 1d). The assembly of colloidal particles is driven by electrophoretic and dielectrophoretic forces generated by the excitation of colloidal particles by electric fields, calculated as follows [58]:

(3) $$\begin{array}{c} F_{EP}=E\varepsilon \zeta \left(\frac{1}{a}+\frac{1}{\lambda_{D}}\right)\pi a^{2} \end{array}$$

(4) $$\begin{array}{c} F_{DEP}=2\pi a^{3}\varepsilon_{l}\frac{\varepsilon_{p}-\varepsilon_{l}}{\varepsilon_{p}+2\varepsilon_{l}}\nabla E^{2} \end{array}$$

where *E*、*ε*、ζ、a、 $\lambda_{D}$ is the electric field strength, the dielectric constant of the droplet, the Zeta potential of the droplet, the average particle size of the nanoparticles, and the electric double layer of the particles. When a voltage *U* is applied between the droplet and the electrode, the contact line is pulled outwards per unit length (Fig. 1c), calculated as [59]: $F_{el}=\varepsilon_{d}\varepsilon_{0}U^{2}/2d$. $\varepsilon_{0}$ is the vacuum dielectric constant, *d* is the thickness of the material deposited on the covering electrode, $\varepsilon_{d}$ is the dielectric constant.

### 2.4 Magnetic Field-Directed Assembly

For droplets containing magnetic colloidal particles, the magnetic volume force generated by applying a magnetic field can act directly on the colloidal particles, while for magnetic droplets, the magnetic volume force (Fig. 1e) generated by deforming or moving the droplets under the action of a magnetic field to change the internal flow field to indirectly affect the deposition of internal particles and complete the directional assembly process can be calculated as follows: [60,61]: $F_{m}=V_{P}\left(M\cdot \nabla \right)B=V_{P}\mu_{0}\left(M\cdot \nabla \right)H$, where, $V_{P}$ represents the volume of the particle; M represents the magnetization of the fluid; B represents the magnetic flux density; H the magnetic field strength; and $\mu_{0}=4\pi \times 10^{-7}$ represents the spatial permeability.

### 2.5 Chemically induced assembly

Beyond the regulation of colloidal particle assembly within evaporating droplets via physical fields, the migration direction of particles can also be controlled by chemical gradients established within the droplet. In particular, diffusiophoresis, driven by chemical concentration gradients, can induce the migration of colloidal particles within the droplet [62]. These gradients arise from solutes such as electrolytes or surfactants. Diffusiophoresis, induced by local solute concentration gradients, has been identified as a key mechanism governing the transport and assembly of colloidal particles. This process inherently involves multi-scale interfacial phenomena and the coupled transport of solutes, fluids, and particles [63]. Guha et al. [16] demonstrated autonomous control of particle distribution within evaporating droplets utilizing salt-induced spontaneous electric fields. They introduced the capillary phoretic number (CP number) to quantify the relative importance of electrophoretic versus convective transport in governing the deposition and pattern formation of colloidal particles. By tuning the CP number, particles can be focused into specific regions or achieve a uniform distribution, thereby enabling the co-assembly of diverse particle populations. Ghosh et al. [64] further explored the application of diffusiophoresis in additive manufacturing. Their work demonstrated that diffusiophoresis can dominate complex evaporative-driven particle assembly processes, offering a novel strategy for controlling particle deposition in multiscale additive manufacturing. Collectively, these studies significantly advance our understanding of directed colloidal assembly within evaporating droplets, encompassing both physical fields and chemical gradients. This work lays the foundation for multi-faceted regulation strategies.

## 3 Applications of field-directed assembly

Contemporary studies categorize external-field-directed assembly into four dominant approaches: acoustic fields, optical fields, electric fields, and magnetic fields. These field-directed approaches provide two critical capabilities: (i) high-throughput fabrication of multiscale architectures through spatiotemporal field programming and (ii) on-demand, dynamic structural reconfiguration through real-time modulation of field parameters. However, each approach exhibits distinct advantages under specific conditions alongside inherent limitations, as summarized in Table 1.

**Table 1:** Summary of the role of the four main types of assembly.

Assembly method	Driving forces	Advantage	Disadvantage
Acoustic assembly	Acoustic radiation force (ARF)	Low energy consumption, High sensitivity, Easy manipulation	Viscous friction generates heat, Acoustic wave thermal effects
	Acoustic streaming flow (ASF)		
Optical assembly	Optical force	Flexible local focusing	High equipment costs
	Marangoni effect		
Electric assembly	Electrophoretic force	High scalability/throughput, Multiscale, The broad applicability of materials	Higher voltage required, safety issues Conductive substrates are required
	Electrowetting		
Magnetic assembly	Magnetophoretic force	Convenient control Low equipment costs, Fast response	Particles/solutions/substrates require magnetic properties
	Negative magnetophoresis		

### 3.1 Acoustic Field-Directed Assembly

**Fig. 2:**
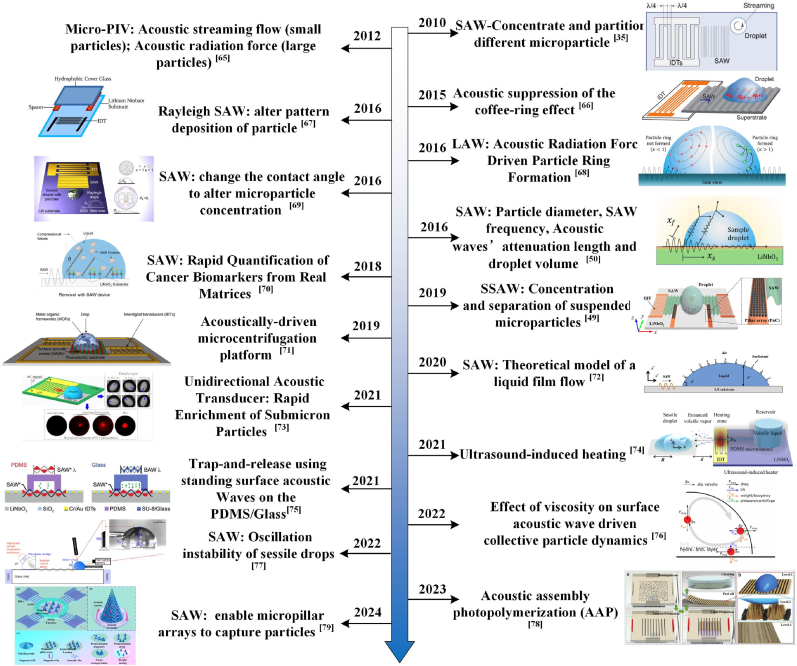
Development of acoustic manipulation assembly [[35], [49], [50], [65], [66], [67], [68], [69], [70], [71], [72], [73], [74], [75], [76], [77], [78], [79]].

**Fig. 3:**
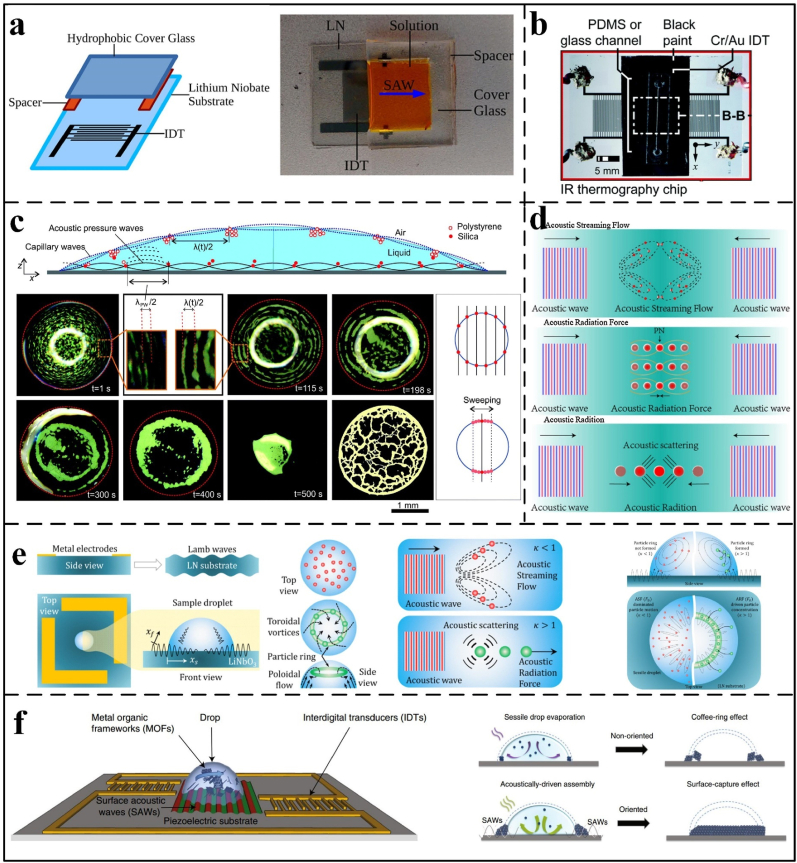
Schematic of particle-oriented self-assembly under surface acoustic wave control. (a) The schematic diagram and physical representation of the experimental system, which consists of a 1 mm rectangular PDMS spacer, illustrate how the single-sided acoustic wave-generating device that produces traveling waves is assembled. Reprinted from Ref. [84]. Copyright 2016 American Chemical Society. (b) We paint a glass-based SAW device black; a symmetrical IDT typically generates the standing wave. Reprinted from Ref. [83]. Copyright 2021, Royal Society of Chemistry. (c) The schematic diagram illustrates the device that manipulates microparticles using the Lamb wave (LW) acoustofluidic platform. Reprinted from Ref. [68]. Copyright 2021 American Chemical Society. (d) The bright-field images of evaporation at different times for droplets containing 4.8 μm polystyrene particles are shown in a schematic diagram of standing waves trapping particles under the action of surface acoustic waves (SAW). Reprinted from Ref. [66]. Copyright 2015 Royal Society of Chemistry. (e) From top to bottom, (1) ASF is generated when the particle diameter is smaller than the SAW wavelength; (2) two counter-propagating saws form SSAW with pressure nodes (PNs), and it is demonstrated that under the action of ARF, particles will drift to the position of PNs; and (3) under the action of two SAWs, ASF significantly hinders particles. Reprinted from Ref. [88]. Copyright 2021 Elserved. (f) An asymmetric acoustofluidic platform controls the directional assembly and activation of a metal-organic framework within the droplet. The graphic illustrates how acoustic waves can assemble and capture particles, resulting in deposition effects during various accumulation phases. Reprinted from Ref. [71]. Copyright 2019 Nature.

Acoustic manipulation, known for its non-contact and high-precision capabilities, is a widely utilized technique in the field-directed assembly of colloidal particles. As shown in Fig. 2, acoustic manipulation methods for colloidal particle assembly have made significant advancements. Electrical signals applied to interdigital transducers (IDTs), as illustrated in Fig. 3a and b, generate surface acoustic waves (SAWs) or bulk acoustic waves, enabling precise particle manipulation within droplets [80,81]. SAWs have found widespread applications in diverse fields such as tissue engineering, high-throughput drug screening, and particularly in analytical chemistry for particle separation, biosensing, and precise particle motion control [82,83]. The unique properties of SAWs render them highly suitable for driving microfluidic systems, offering advantages including low energy consumption [35], rapid response times [82], high sensitivity [83], and excellent biocompatibility [82,83]. Crucially, the practical implementation of acoustic manipulation relies on precise control mechanisms. Standing acoustic waves generate pressure nodes that precisely control particle positioning during droplet evaporation, resulting in concentric circular deposition patterns [66]. Modulating the frequency of these waves allows for precise adjustment of particle deposition density, facilitating tailored assembly morphologies for applications such as biosensing [84]. Furthermore, this standing wave-based approach exhibits robustness against variations in particle shape and fluid composition, making it highly suitable for particle manipulation in biological and chemical analytical assays [[85], [86], [87]].

Based on their operational mode, acoustic waves can be classified as standing or traveling. Standing waves, generated by symmetrically positioned IDTs (Fig. 3a and b), enable localized particle trapping at pressure nodes within droplets [88]. On the other hand, traveling waves simplify the device layout, requiring only one IDT device (Fig. 3a) to apply the force to the particles [89]. Mampallil et al. [66] demonstrated that SAWs suppress the CRE by forming standing acoustic and capillary waves, as illustrated in Fig. 3c. These wave nodes trap particles, resulting in closely spaced concentric patterns. Acoustic pressure wave nodes predominantly trap heavier particles (silica, 1.9 g/cm^3^), while capillary wave nodes capture lighter ones (polystyrene, 1.05 g/cm^3^). Droplet dimensions dictate capillary wave characteristics, such as wavelength, which in turn influence the precision of particle patterning. As evaporation progresses, the spacing between concentric polystyrene patterns widens, reducing the number of rings [66]. Han et al. [90] developed an SSAW platform to investigate how frequency modulation affects droplet contact angle and particle dynamics during separation and concentration. Their findings reveal that the dominance of acoustic streaming flow (ASF) and acoustic radiation force (ARF) shifts with changes in the contact line. Frequency and contact angle evolution critically influence particle control, such as in Fig. 3d. Peng et al. [73,91] employed a unidirectional IDT to generate SAWs, driving internal droplet flow and rapidly concentrating submicron particles at the center. Size-selective particle manipulation enhances micron-scale particle enrichment, advancing diagnostic assays.

Furthermore, particle alignment and aggregation can be precisely tuned by adjusting wave frequency, type, and particle size. Destgeer et al. [50,69] investigated the size-dependent migration of polystyrene particles under surface acoustic waves (SAWs), revealing distinct partitioning behaviors. Specifically, SAWs generate ASF and ARF, with ARF scaling with particle diameter, enabling size-based particle separation into different regions. Building on this, Destgeer et al. [68]^,^ employing Lamb waves (LWs), showed that increasing particle concentration shifts dominance from ARF to ASF, yielding uniform distributions (Fig. 3e). The characterization of these distinct ARF-dominated and ASF-dominated regions was further developed by Nam et al. [92], who explained how SAWs can be used to manipulate particle mixing, enrichment, and separation, capabilities essential for rapidly enriching small-scale particles required in chemical and biological detection. Song et al. [76] altered the droplet viscosity and particle size, observing three distinct states: dispersion, central cavity formation, and central convergence. These states arise because larger particles (≥10 μm) cannot follow the acoustic streaming flow streamlines, creating a stagnation region (or 'forbidden zone'). In contrast, smaller particles (≤1 μm) can readily migrate along these streamlines. Ahmed et al. [71] proposed a SAM-powered microcentrifugation platform to facilitate the extraction of oriented metal-organic frameworks (MOFs) crystals. During gradual evaporation, a weak convective flow persists within the droplet. This flow transports solute molecules to the contact line, resulting in the formation of a ring of crystals (Fig. 3f), which exhibit no long-range ordering. As shown in Fig. 3f, the superlattice structure creates a homogeneous and continuously layered monolayer when the Rayleigh surface acoustic wave (SAW) is activated. As a result, the solute molecules begin to flow rapidly and turbulently in the direction of the contact line. Weaker CRE in continuous stacking permits the solute to create a single layer of homogeneous deposition.

**Fig. 4:**
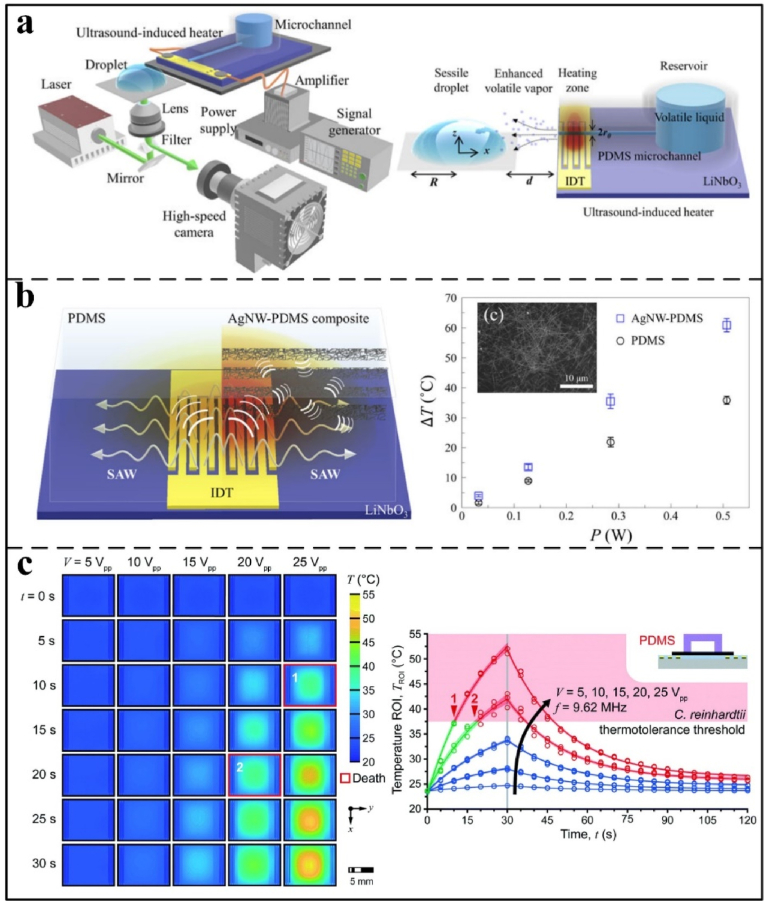
(a): Ultrasonic-induced heating enhances the Marangoni effect inside the droplet and promotes evaporation. b) The picture on the left shows how acoustic waves heat PDMS and AgNW-PDMS composites when used as the ultrasonic absorbing layer. The picture on the right shows how the temperature rises when ultrasonic waves are used at 29 MHz. Reprinted from Ref. [94]. Copyright 2021. (c) Under the stimulation of a 10 MHz acoustic wave, the temperature of the PDMS substrate gradually increased with time. Reprinted from Ref. [81]. Copyright 2021 Royal Society of Chemistry.

During the acoustic assembly, the temperature of the droplet increases due to piezoelectric substrate oscillation, resulting in undesirable heating that can limit its applicability. Das et al. [93] attributed this heating to mechanical stress from substrate vibrations and viscous friction induced by acoustic energy dissipation within the droplet. Consequently, they found that the temperature rises linearly with SAW power. Cha et al. [74] employed a silver nanowire-PDMS (AgNW-PDMS) composite as an acoustic-absorbing layer to enhance Marangoni flow by increasing heat absorption, thereby accelerating evaporation and mixing in high-viscosity droplets (Fig. 4a and b). In contrast, Akther et al. [94,95] showed that PMMA substrates reduce acoustic wave absorption, minimizing indirect droplet heating. Cui et al. [75] investigated acoustic wave devices composed of polydimethylsiloxane (PDMS) and glass (Fig. 3b). They found that cell and particle trapping is effective at low frequencies.

Furthermore, glass-based SAW devices have been shown to enable particle or cell manipulation at high frequencies. However, PDMS devices heat up rapidly under acoustic excitation; this excessive heating can destroy biological cells and other matter (Fig. 4c). For example, Mehmood et al. [81] reviewed the advantages and disadvantages of surface acoustic wave thermal effects for medical applications. While the thermal effect can be utilized in applications such as polymerase chain reaction, thermal cycler, and blood coagulation, handling viable biological cells necessitates avoiding excessive heating to prevent thermal inactivation or damage.

Acoustic field-directed assembly and control technology offers advantages such as wide adaptability, low energy consumption, rapid assembly, and strong scalability. It can control colloidal particles in numerous dispersed systems without being limited by material properties such as conductivity and magnetism. However, it still faces challenges, such as difficulty in achieving high-precision assembly control and high dependence on substrate material properties. These challenges can be addressed through strategies such as substrate surface modification to mitigate particle agglomeration or simulation-guided optimization of the acoustic field for more precise manipulation of micro- and nano-particles.

### 3.2 Optical field-directed assembly

**Fig. 5:**
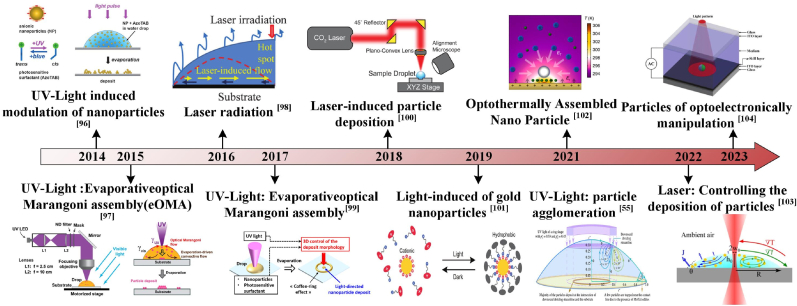
Development of optical manipulation assembly [[55], [96], [97], [98], [99], [100], [101], [102], [103]].

**Fig. 6:**
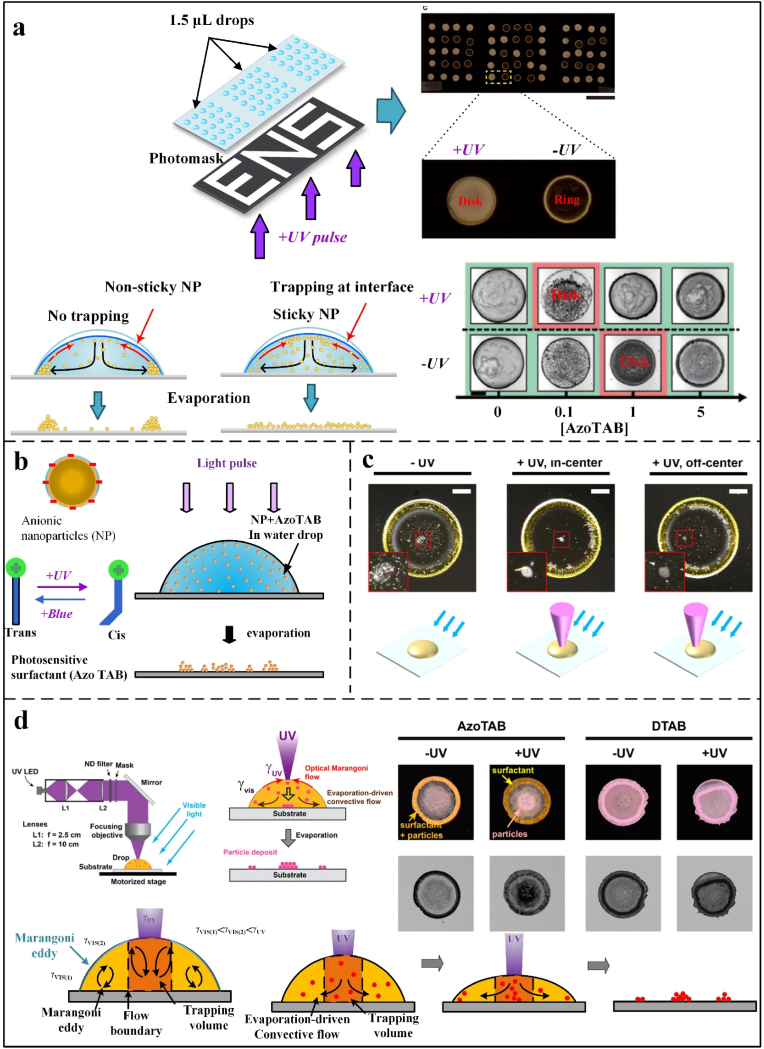
Optical-induced directional assembly of particles. (a, b) Ring/disk deposition of droplets under optical control: when the Marangoni flow (red arrow) is insufficient to compensate for the evaporation-driven capillary flow (black arrow), the particle will move towards the contact line, and the concentration of optical and AzoTAB will also affect the particles' deposition shape [96]. Copyright © 2017 American Chemical Society. (c) The impact of UV beam irradiation and its position on droplet evaporation deposition is examined. Reprinted from Ref. [99]. Copyright 2017 American Chemical Society. (d) The schematic diagram illustrates the impact of UV light and the presence or absence of photosensitive particles on the state of particle deposition during droplet evaporation. It also compares the experimental results with the gradient of azo concentration towards the contact line and the optically induced Marangoni flow towards the UV light. The boundary between the two circulating flows defines a region where the Marangoni at the edge of the gurney vortex traps particles. Reprinted from Ref. [97]. Copyright 2016 American Chemical Society.

Optical forces arising from gradient and scattering forces exerted by a tightly focused laser beam were first demonstrated for manipulating microparticles (25 nm–10 μm) in 1986 [105]. Building on this principle, subsequent research has refined optical manipulation techniques to enhance assembly precision and flexibility [106,107], as illustrated in Fig. 5. Localized optical stimulation facilitates the nucleation, diffusion, and growth of colloidal particles, enabling directed multi-dimensional assembly critical for applications such as biosensing and particle separation [44,100], the manufacturing of photonic crystals and smart sensors [108,109], or the improvement of energy conversion performance and power density resulting from the formation of local concentration or temperature gradients caused by photothermal effects. A typical application involves modulating optical forces to assemble colloidal particles during droplet evaporation by illuminating the droplet to induce photothermal effects. These effects generate Marangoni flows that alter the internal flow field and drive the aggregation of particles. This controlled flow enables tailored particle distribution within the droplet, resulting in specific deposition patterns post-evaporation (as shown in Fig. 6a). Alternatively, optical manipulation can directly modify photosensitive surfactants within the droplet, inducing constant-temperature internal flows that direct particle aggregation [96,99]. These capabilities are particularly valuable in analytical chemistry for applications such as biosensing and particle chromatography [[110], [111], [112]].

Optical field-directed assembly can directly manipulate photosensitive particles or indirectly influence particle motion by altering internal droplet flows via photothermal effects. Achieving uniform deposition requires thoroughly balancing capillary and laser-induced flows by precisely adjusting laser parameters, including power, exposure time, and beam diameter [55,98]. Additionally, steering the laser spot or employing masks with varied transparency enables the creation of complex deposition patterns, enhancing applications such as laser-induced particle etching for surface analysis. Li et al. [113] demonstrated that focused infrared lasers enhance Marangoni flow through photothermal effects, thereby accelerating particle movement and preventing contact line pinning, which promotes uniform deposition. Additionally, Goy et al. [103] developed a model predicting Marangoni flow coverage, linking temperature gradients to improved particle self-assembly regularity. Gao et al. [114] optimized particle transport efficiency by inducing asymmetric droplet deformation, thereby manipulating Marangoni flow. Thokchom et al. [115] utilized particle image velocimetry (PIV) to demonstrate that surface temperature distributions dictate Marangoni-induced convection patterns, as heating symmetry affects vortex formation and uniformity of particle deposition. These advancements underscore the importance of thermal control in optical manipulation for analytical applications, such as surface patterning.

Unlike photothermal effects, the use of direct optical control of photosensitive surfactants enables dynamic regulation of CRE by optically tuning particle stickiness at liquid-air interfaces without altering droplet temperature. Anyfantakis et al. [96] mixed negatively charged polystyrene nanoparticles with photosensitive surfactant (AzoTAB) via electrostatic/hydrophobic interactions. UV-induced polarity changes in AzoTAB affect its adsorption on particles (Fig. 6a and b), governing particle viscosity at interfaces. By modulating UV intensity and area, surfactant concentration, and nanoparticle size and shape (Fig. 6c), their study [99] demonstrated control of sub-30 nm particles via evaporative optical Marangoni assembly (eOMA), thereby enhancing optical-driven precision and deposition scale (Fig. 6b and c). Indeed, it is possible to regulate the assembly of particles using a light field without changing the droplet temperature. Varanakkottu et al. [97] dissolved the photosensitive surfactant in the regulated droplet. They utilized optical methods to generate photochemical surface-tension driven interfacial flow (optical Marangoni flow) and induce internal flow via a high concentration of the surfactant, guiding particle deposition. The droplet interior composition had little effect on this process, enabling organized particle deposition on the substrate in arbitrary patterns. This approach proposes a simple-to-operate yet versatile strategy (Fig. 6d).

In summary, the optical field-directed assembly offers a means for versatile control of particle deposition through direct force application and modulation of the flow field. While photothermal effects offer precision via intense lasers, the resultant heat poses significant risks to heat-sensitive samples, particularly due to their vulnerability to thermal damage, such as live cells in biosensing. Photosensitive surfactants provide a temperature-independent alternative; however, the concentration of which must be carefully optimized to avoid altering the solution properties. Future efforts should focus on minimizing thermal damage through refined laser parameters or integrating optical methods with other field-directed approaches to enhance precision and biocompatibility in analytical applications.

### 3.3 Electric field-directed assembly

**Fig. 7:**
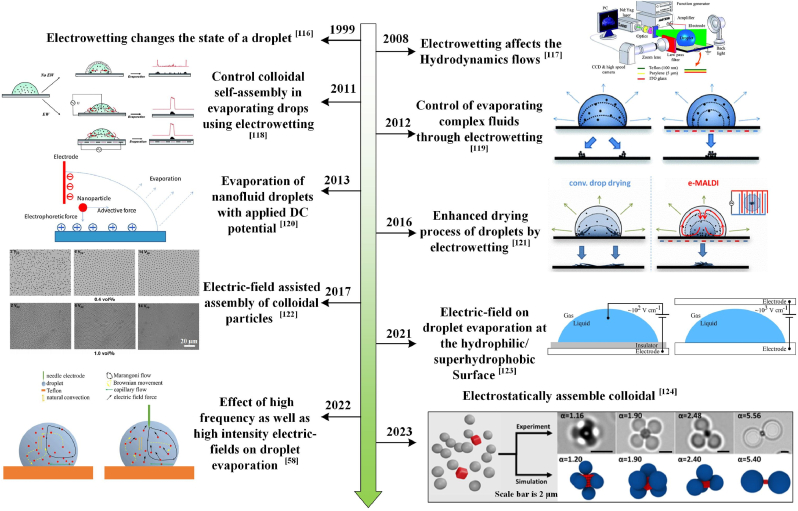
Development of electric-field manipulation assembly [[58], [116], [117], [118], [119], [120], [121], [122], [123], [124]].

Within the droplets, colloidal particles frequently acquire surface charges through mechanisms such as surface group ionization, electrostatic attraction, or chemical reactions. Upon applying an electric field, oppositely charged electrodes attract these particles, inducing directional migration. Tailoring the field's voltage and frequency enables rapid and large-scale colloidal assembly, which is critical for particle separation and biosensing [125]. Electric fields enable diverse colloidal assembly configurations, as illustrated in Fig. 7. Through electrophoresis [58,126,127] and particularly via electrowetting effects [128,129], the applied field disrupts the equilibrium between the droplet's contact angle and contact line [120,121]^.^ Specifically, electrowetting helps overcome contact line pinning, reduces contact angle hysteresis, and induces contact line movement when the electrowetting-induced force exceeds the pinning force threshold. This disruption, driven by interfacial potential differences, reduces the contact angle and expands the contact area, directly influencing particle motion or indirectly altering the internal flow field. Concurrently, significant changes in droplet shape [44,120] modify the wetting state, evaporation rate, and flow dynamics, ultimately shaping the particle deposition pattern.

**Fig. 8:**
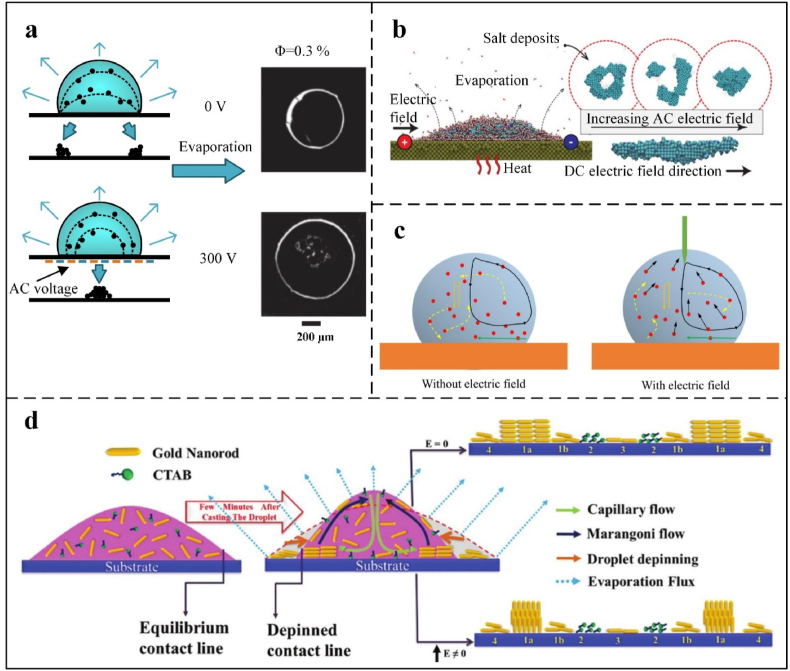
(a): Normally, the droplet evaporates with a fixed contact line; when subjected to an electric field, the droplet's contact line moves, changing the particle's deposition position. Reprinted from Ref. [118]. Copyright 2012, Royal Society of Chemistry. (b) The shapes of the particles that remain after saline nanodroplets evaporate under alternating electric fields (AC) and direct electric fields (DC) were compared. Reprinted from Ref. [129]. Copyright 2016 American Chemical Society. (c) This section discusses the flow field distribution within a droplet, both with and without the presence of an electric field. Reprinted from Ref. [58]. Copyright 2022. (d) A schematic illustration of the evaporation of dispersed droplets containing Au-NR on a hydrophilic substrate, with and without a DC electric field, shows that the intervention of an electric field alters the final deposition mode of the internal particles after the droplet evaporates. Reprinted from Ref. [133]. Copyright 2022, Royal Society of Chemistry.

Electric fields are typically classified as alternating current (AC) or direct current (DC), each offering distinct advantages for directional particle assembly [122]. In AC fields, the electric force per unit length on the contact line is given by *f*_el_ = *ε*_d_*ε*_0_*U*^2^/2*d*, where *d* is the dielectric substrate thickness, ε_d_ its dielectric constant, *ε*_0_ the vacuum permittivity, and *U* is the applied voltage. This force modulates the contact line's dynamics: when *U* exceeds a critical threshold, it overcomes pinning, initiating movement [118]. This shift indirectly influences colloidal particles by altering the droplet's internal flow field, a key mechanism for controlling deposition patterns in analytical assays. In AC fields, modulating the voltage amplitude controls particle aggregation or dispersion during evaporation, suppressing the CRE [119]. As shown in Fig. 8a, Eral et al. [118] demonstrated that AC fields (several Hz to kHz) inhibit CRE across colloidal and DNA solutions by altering the droplet's flow field to prevent particle migration to the contact line. Zhang et al. [116] modeled the evaporation of saltwater nanodroplets on platinum (Fig. 8b), demonstrating that electrowetting induces diverse deposition modes, with low-frequency, high-strength AC fields altering particle shapes. A threshold field strength of 0.006 V/Å marks the transition from ring-like to clump deposition, enabling CRE suppression without the need for additives [121]. Al Harraq et al. [130] studied the co-assembly behavior of non-active isotropic passive particles and metal-coated active particles using an alternating electric field method. They revealed the role of non-reciprocal interactions in colloidal assembly and demonstrated that the reciprocity of interactions can be controlled by designing active particles and their motion trajectories. By controlling the proportion of active particles, programmable fluctuations and reconstruction of aggregates can be achieved.

Conversely, DC fields deform sessile droplets by modifying interfacial potential, surface tension, and contact angle, as described by cos*θ*(*V*) = cos(*θ*_0_) +0.5*ε*_r_*ε*_0_*V*^2^/*γ*_lg_*d*, where ε_r_ is material permittivity, *ε*_0_ is space permittivity, γlg is liquid-gas interface surface tension, and d is insulating film thickness [23]. Combined with dielectrophoretic forces, this facilitates the precise positioning of particles. Nanoparticles exhibit “stick-slip” behavior, characterized by intermittent contact line motion and stepwise deposition [131,132]. As the DC field persists, particles deposit along the slowly retracting contact line, enhancing uniformity in diagnostic assays [23,118]. Unlike AC electric fields, which modulate particles through flow fields, DC electric fields achieve directional deposition primarily through droplet deformation and electrophoresis.

Electric field strength and frequency critically influence droplet evaporation and particle control. Below 10 Hz (for millimeter-sized droplets), unstable flows yield spot deposits during quasi-static contact line retraction. At 10–10^3^ Hz AC, this causes periodic electric stress on the contact line, sending capillary waves to the drop-top [112]. Ko et al. [117] decomposed AC wetting flow into low-frequency (interfacial vibration-driven) and high-frequency zones (conductivity/electrode-dependent). High-frequency AC (>10^4^ Hz) generates both ohmic and displacement currents. Electric fields penetrate liquids to generate ohmic and displacement currents when high-frequency alternating current (>10^4^ Hz) is applied. Chen et al. [58] investigated the impact of high-frequency and high-intensity AC electric fields on droplet evaporation and wetness (Fig. 8c). They found that the evaporation rate increased gradually with increasing voltage frequency (0–2 kHz). Changing the droplet profile by increasing or decreasing the electric field improves both the droplet surface and the internal flow field. Experimental results suggest that high-frequency flow is more sensitive to electrodeposition and solute concentration. Combining and modifying high- and low-frequency AC reduces deposition area [118]. Zaibudeen et al. [133] investigated the effect of a DC electric field on the evaporation of stationary gold nanofluid droplets. They found that the droplets form coffee rings with different nanoparticle aggregation regions during the evaporation process, that the gold nanorods show different arrangements at the edges of the coffee rings when a DC electric field is applied, and that the presence of the electric field causes the gold nanorods to be arranged in the direction of the electric field at the edges of the coffee rings. In contrast, the gold nanorod orientations in the non-coffee ring edge region of the gold nanorods are not affected by the electric field (Fig. 8d). The choice of intensity and frequency of the electric field, whether AC or DC, governs droplet evaporation and particle dynamics.

Beyond field parameters, solution pH, substrate wettability, and electrode geometry also affect electric field-directed assembly. Ramírez-Ramírez et al. [134] simulated pH-dependent colloidal crystal aggregation and dispersion via electrostatic forces. Optimized waveforms and tailored electrodes enhance precision in particle motion during evaporation [112,116]. Substrate hydrophilicity or hydrophobicity [44,118] and field positioning [119,120] further influence deposition. Electrowetting excels across diverse colloidal suspensions, benefiting analytical applications [121]. Electric field manipulation can be categorized as either intrusive or non-intrusive, depending on the electrode configuration [122]. Non-intrusive methods (cross-electrode or radial electroosmotic setups) avoid direct droplet contact, preserving sample integrity. Operating at low voltages, this approach is suitable for biological samples, supporting diagnostic assays without compromising tissue viability [122].

Electric field-directed assembly is compatible with a wide range of materials, including conductive, semiconductor, and dielectric particles, making it highly versatile across multiple fields such as electronics, photonics, and biotechnology. However, the electric field-directed assembly also faces particular challenges. The primary issue is the potential for particle aggregation, particularly when handling high concentrations or charged particles, which may lead to undesirable aggregation and disrupt the desired assembly patterns. Another challenge is the need for high voltages in specific applications, which may result in dielectric breakdown or thermal effects, potentially damaging the assembled structures or substrate. Optimizing electric field parameters and assembly conditions (such as particle suspension concentration and pH) can help reduce aggregation. Using dielectric barriers or insulating layers can also prevent dielectric breakdown. Additionally, developing hybrid methods that combine EFDA with other assembly techniques (such as self-assembly or magnetic field-assisted assembly) can enhance the robustness and versatility of the assembly process. These combined methods can leverage the advantages of each technique, overcoming the limitations of EFDA alone and paving the way for more efficient and reliable manufacturing of advanced nanostructured materials.

### 3.4 Magnetic Field-Directed Assembly

**Fig. 9:**
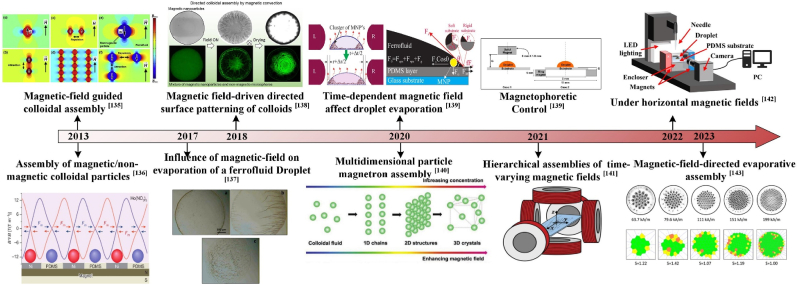
Development of magnetic manipulation assembly [[135], [136], [137], [138], [139], [140], [141], [142], [143]].

The magnetic field is one of the more widely used manipulation outfields, as shown in Fig. 9, and plays a vital role in the manipulation process of directed assembly. Magnetic fields influence colloidal assembly by generating magnetic gradient forces and torques, which act directly on magnetic particles or indirectly modify droplet dynamics, including internal flow fields and contact line behavior [144]. Standard magnetic control devices fall into hybrid magnets, micro-integrated magnets, and external macro-magnets. When sufficiently proximate, magnetic fields induce forces and torques that enable the capture, assembly, mixing, and delivery of micro- and nano-objects [144]. By regulating the magnetic field, anisotropic particles can be assembled into structures with good spatial order [145,146]. The primary determinants of droplet evaporation and ultimate deposition morphology are the concentration of nanoparticles, the direction and strength of the applied magnetic field, and the type of substrate material.

**Fig. 10:**
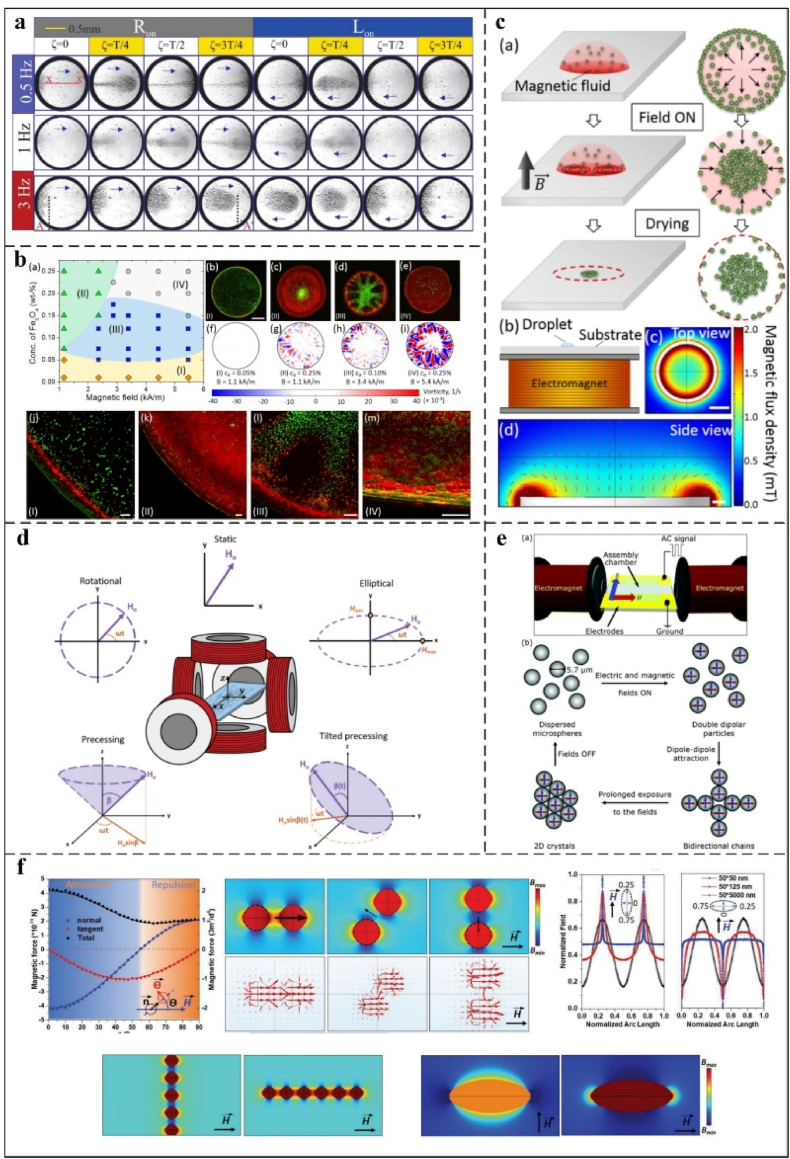
(a): Effect of Magnetic Field Frequency on Particle Assembly in Evaporating Ferrofluid Droplets. Reprinted from Ref. [139]. Copyright 2020 Royal Society of Chemistry. (b) Illustrates the internal particle distribution of the mixed solution after evaporation and drying [138]. (c) This section presents perspective and top-down schematic diagrams that illustrate the evaporation process of a solution that contains a mixture of magnetic (red) and nonmagnetic (green) particles, all under the influence of a uniform magnetic field. Reprinted from Ref. [138]. Copyright 2018 American Chemical Society. (d) A three-dimensional magnetic field control device allows for spatial control. Reprinted from Ref. [141]. Copyright 2021 Royal Society of Chemistry. (e) Electromagnets control the formation of 2D crystals. (f) The schematic diagram shows the magnetic force between magnetic nanoparticles and their straight and curved parts, which was found through simulations and the solution of an analytical equation. It is possible to form bidirectional chains and generate additional morphological structures. Reprinted from Ref. [152]. Copyright 2016 Royal Society of Chemistry. (f) The schematic diagram illustrates the magnetic force between magnetic nanoparticles and their regular and tangential components, as determined by simulation calculations and an analytical equation solution. Reprinted from Ref. [140]. Copyright 2020.

Magnetic interactions, defined as forces between magnetic dipoles, play a pivotal role in controlling the orientation and spatial arrangement of anisotropic particles, thereby facilitating precise crystal formation and effective assembly. Ferrofluids, composed of magnetic nanoparticles, a base fluid, and surfactants, exhibit exceptional responsiveness to magnetic fields modulated by field strength and frequency [147,148]. Shyam et al. [139] experimented to discover how a drop of ferrofluid evaporates on the soft matter when a magnetic field changes over time. They indicated that the overall lifetime of the evaporating ferrofluid droplet depends on the movement of the magnetic nanoparticles. At lower magnetic field frequencies, particles align into chains along field lines (Fig. 10a), while at higher frequencies, they aggregate centrally due to insufficient time to reach the contact line. Saroj et al. [149,150] reported the effect of the magnetic field strength on the evaporating and drying performances of ferrofluid drops. The distance between the magnet and the droplet changes the field strength. Their research indicated that as the magnetic field strength increases, the receding contact angle increases and the initial contact angle reduces. The concentration of nanoparticles near the contact line is lower, thereby reducing the frictional resistance to the motion of the contact line.

Alternating or static magnetic fields can also influence and control the movement of particles suspended in sessile droplets. To achieve directional migration of internal particles by modulating the magnetic field and changing variables such as magnetic field strength, gradient, or concentration of magnetic nanoparticles [137,143]. Lee et al. [138] utilized magnetostatic microconvection to assemble nonmagnetic particles in magnetic fluids into predefined patterns (Fig. 10b and c), thereby reversing particle transport from the center to the edge and diversifying assembly strategies. Jadav et al. [137] employed a static magnetic field to investigate the influence of magnetic field magnitude and orientation on the evaporation dynamics of an aqueous ferrofluid deposited on glass substrates. The applied magnetic field imparts a torque on the magnetic particles, given by $S=mxH=m_{s}V_{p}Hcos\theta$, (where m is the magnetic moment, m_s_ is the saturation magnetization of the particle, *V*_*p*_ is the particle volume, and *H* is the applied magnetic field), thereby controlling particle rotation, translational motion, and assembly during evaporation. Through research, Liu et al. [142] found that droplet concentration and magnetic field strength affect the changes in contact angle and contact line during droplet evaporation. Spatafora-Salazar et al. [141] show that time-varying magnetic fields produce bipolar interactions that vary in time and space, which can modulate superparamagnetic colloids from chain to sheet assembly, enabling the assembly of particles from one to two or even multi-dimensional, providing a basis for the directed assembly of hierarchical particles (Fig. 10d).

In addition to applying a static magnetic field, changing the type of magnetic field or combining it with other external fields can improve the accuracy of particle orientation assembly. Li et al. [140] demonstrated that magnetic fields can magnetize superparamagnetic and ferromagnetic nanoparticles, with the resulting magnetization governed by the spatial uniformity of the field. The study also highlights the need to integrate the magnetic response mechanism with other responses to enhance the directional assembly of nanomaterials. Bharti et al. [151] noted (Fig. 10e) that a magnetic field can adjust the dipole interaction between nano- and micro-particles. An alternating current (AC) electric field and a uniform magnetic field facilitate the assembly of coatings, dynamically reconfigurable particle networks, and bidirectionally moving active structures. An external magnetic field can magnetize superparamagnetic and ferromagnetic nanoparticles through induced magnetic dipoles. They also noted that changing the type of magnetic field can make magnetic particles stick together (Fig. 10f) [139].

An equally important influence on the directed assembly process is the substrate on which the droplets are cemented. Guba et al. [152] investigated the impact of magnetic fields on the contact line and contact angle of an aqueous ferrofluid deposited on substrates spanning hydrophilic to hydrophobic properties. Their findings revealed that on hydrophilic silicon wafers, the contact line remained largely unaltered throughout droplet extension under magnetic field exposure, whereas the contact angle exhibited more significant variation. When tested on hydrophobic silica surfaces under identical conditions, the contact angle demonstrated minimal variation. Kaushal et al. [153] studied the kinetics of droplet evaporation on different substrates (hydrophilic and superhydrophobic). Using infrared thermal imaging, they saw the magnetocaloric phenomenon inside the droplet. This demonstrated that the magnetic field generates a temperature difference within the droplets, initiating the magneto-solute Marangoni circulation. The magnetophoretic or negative magnetophoretic effect generated under the action of a magnetic field can regulate the movement of both magnetic and non-magnetic particles, thus accomplishing oriented assembly on demand. Magnetic particles move to places with strong magnetic fields in positive magnetophoresis. Negative magnetophoresis technology is non-invasive, doesn't add extra heat to the droplet, and can be used to move biological particles. Saroj et al. [154] reported the negative magnetophoretic effect of non-magnetic particles in ferrofluid evaporation. They utilized solid magnets and ring magnetism to alter the distribution of the magnetic field, adjusted the distance between the droplet of interest and the magnetic field to modify its strength, and calculated the magnetic field. They demonstrated the ability to control the negative magnetophoretic effect on particle deposition. This enables the selective deposition of antimagnetic polystyrene microspheres.

Magnetic Field-Directed Assembly (MFDA) offers unique advantages, enabling remote control of magnetic or magnetically responsive particles without physical contact, guiding their arrangement and assembly to facilitate the construction of complex three-dimensional structures. Magnetic field strength and direction are easily adjustable, enabling precise control over the assembly process. Additionally, MFDA exhibits strong environmental adaptability, capable of operating in various media, with a gentle operational process that helps maintain the integrity of the assembled structures. However, MFDA also faces challenges, such as limited control over non-magnetic or weakly magnetic particles, restricted magnetic field penetration, and uneven aggregation and assembly structures caused by magnetic dipole interactions between particles. To address these issues, researchers have proposed methods such as magnetic modification of particles, using multipole magnetic field sources or optimizing magnetic field source layout, precisely controlling magnetic field strength and change rates, and optimizing particle concentration and dispersion states to enhance manipulation capabilities, improve assembly efficiency, and achieve uniform and stable assembly.

With the increasing demand for colloidal particle assembly and structural complexity, a single assembly strategy is no longer sufficient. Consequently, the collaborative coupling of multiple control methods is essential to overcome the limitations inherent in singular approaches. Therefore, control methods are developing in the direction of composite coordination of multiple physical fields (such as sound, light, electricity, and magnetism) to overcome single-field limitations and achieve more accurate control of colloidal particles. For example, Shen et al. [155] developed acoustic electric tweezers technology by combining acoustic radiation force and dielectric electrophoresis force, which achieved independent synchronous manipulation of multiple colloidal particles by regulating surface acoustic waves and local electric fields. The physicochemical coupling strategy enables precise regulation of the anisotropic assembly process involving magnetic-field-induced Fe_3_O_4_ nanoparticles and mesoporous silica. Thereby controlling the branching density, length, and roughness of the nano chains, which can be used to prepare branched magnetic mesoporous nano chains with adjustable surface roughness (Fe_3_O_4_@mSiO_2_) [156]. In addition, Li et al. [157] noted that the combination of optical and acoustic forces can form multiple potential wells, enabling the stable capture and precise positioning of particles with varying refractive indices. Microstructure engineering [158] and responsive material design [159] also provide additional strategies for achieving high precision, high flexibility, and fast response in collaborative assembly manipulation.

## 4 Challenges and outlooks

Over the past two decades, the study of evaporation dynamics in colloidal droplets has developed into a multidisciplinary field that integrates fluid mechanics, soft matter physics, materials science, and analytical chemistry. This process applies to a wide range of advanced technologies, including large-scale, high-precision printing, biological cultivation, and chemical analytical detection. However, the mesoscopic environment within the droplet poses significant challenges for achieving precise control over diverse particle populations. At this scale, complex hydrodynamics (e.g., internal flows, capillary flows), interparticle interactions (van der Waals, electrostatic, steric), and evaporation-driven non-equilibrium processes are intricately coupled, rendering particle transport trajectories highly complex and difficult to predict. A major scientific challenge lies in unraveling these intricate interactions and flow mechanisms to develop predictive models for particle behavior. Externally applied fields (acoustic, optical, electric, and magnetic) and their nonlinear coupling provide tunable pathways to steer non-equilibrium particle transport during evaporation-driven phase separation, facilitating programmable assembly of hierarchical architectures from 2D monolayers to 3D superstructures. However, the mechanistic understanding of how these external fields influence particle dynamics at molecular and colloidal scales remains incomplete, posing a critical scientific frontier. Multiscale theoretical frameworks, coupled with precision manipulation strategies, become imperative in this inherently stochastic, non-equilibrium regime dominated by competing interactions across molecular to colloidal length scales. Consequently, the adoption of high-resolution, sustainable visualization techniques is crucial for elucidating particle motion patterns and optimizing corresponding control methods. Furthermore, with the rapid advancement of artificial intelligence (AI), strategies for the directed assembly of colloidal particles must evolve toward intelligent, efficient paradigms, leveraging rapid droplet composition analysis to achieve precise matching and high-throughput preparation tailored to specific applications.

This review evaluates four primary external field manipulation methods—acoustic, optical, electric, and magnetic, and examines recent advancements in the directed assembly of colloidal particles within evaporating droplets. External fields impose spatially anisotropic forces to regulate particle trajectories during droplet evaporation. Additionally, it addresses the challenge of uneven particle deposition caused by the CRE, thereby improving the reproducibility, precision, and utility of colloidal deposition processes. Central to this review are the mechanisms and applications of these four techniques, which control particle deposition through various means such as pinning, trapping, or disrupting internal flows. Field-directed assembly offers several advantages, including minimal reliance on chemical additives, which preserve the droplet's intrinsic properties while achieving targeted particle orientation. Consequently, external field-controlled colloidal assemblies hold significant potential for future development. Despite substantial progress, challenges persist across these methods. For instance, electric field manipulation is less effective on hydrophilic surfaces and requires conductive substrates, restricting its applicability to colloidal particles smaller than 100 nm. Magnetic manipulation necessitates the use of magnetic particles, while optical methods, although offering high resolution, often require substantial power and may pose safety concerns due to their energy consumption. Moreover, a scientific challenge persists in fully understanding the physical principles governing field-particle interactions, which is essential for optimizing these techniques for broader applicability and efficiency. Addressing these limitations, particularly energy consumption and safety concerns, is critical for wider adoption.

Despite these challenges, the field-directed assembly has already demonstrated significant impact in diverse fields, including inkjet printing, low-cost medical diagnostics, bioengineering, and chemical manufacturing. Its capacity for physical control with minimal alteration to droplet properties sets it apart from traditional nano-assembly techniques. We anticipate that directional assembly will progress to large-volume, large-area, and high-speed assembly. Future developments are expected to involve hybrid control strategies integrating multiple external fields, thereby enabling more versatile and precise colloidal particle assembly. Overcoming these scientific challenges through advanced theoretical and experimental approaches will be pivotal in unlocking the full potential of these future advancements.

## CRediT authorship contribution statement

**Yongqing He:** Writing – original draft, Methodology, Investigation, Conceptualization. **Jian Liu:** Writing – review & editing, Investigation. **Xukai Yang:** Writing – review & editing, Investigation. **Jianzhi Yang:** Writing – review & editing, Methodology, Conceptualization. **Feng Jiao:** Writing – review & editing, Supervision, Methodology, Funding acquisition.

## Declaration of competing interest

The authors declare that they have no known competing financial interests or personal relationships that could have appeared to influence the work reported in this paper.

## Data Availability

Data will be made available on request.
